# Comparison of low and high dose ionising radiation using topological analysis of gene coexpression networks

**DOI:** 10.1186/1471-2164-13-190

**Published:** 2012-05-17

**Authors:** Monika Ray, Reem Yunis, Xiucui Chen, David M Rocke

**Affiliations:** 1 Division of Biostatistics, School of Medicine, University of California, Davis, CA, USA

## Abstract

**Background:**

The growing use of imaging procedures in medicine has raised concerns about exposure to low-dose ionising radiation (LDIR). While the disastrous effects of high dose ionising radiation (HDIR) is well documented, the detrimental effects of LDIR is not well understood and has been a topic of much debate. Since little is known about the effects of LDIR, various kinds of wet-lab and computational analyses are required to advance knowledge in this domain. In this paper we carry out an “upside-down pyramid” form of systems biology analysis of microarray data. We characterised the global genomic response following 10 cGy (low dose) and 100 cGy (high dose) doses of X-ray ionising radiation at four time points by analysing the topology of gene coexpression networks. This study includes a rich experimental design and state-of-the-art computational systems biology methods of analysis to study the differences in the transcriptional response of skin cells exposed to low and high doses of radiation.

**Results:**

Using this method we found important genes that have been linked to immune response, cell survival and apoptosis. Furthermore, we also were able to identify genes such as BRCA1, ABCA1, TNFRSF1B, MLLT11 that have been associated with various types of cancers. We were also able to detect many genes known to be associated with various medical conditions.

**Conclusions:**

Our method of applying network topological differences can aid in identifying the differences among similar (eg: radiation effect) yet very different biological conditions (eg: different dose and time) to generate testable hypotheses. This is the first study where a network level analysis was performed across two different radiation doses at various time points, thereby illustrating changes in the cellular response over time.

## Background

Humans are exposed to ionising radiation from natural and medical (either therapeutic or occupational) sources. While occupational related exposure involves low doses of radiation (< 0.5 Gy), therapeutic doses of radiation can be moderate to high (> 1 Gy). Medical procedures, such as diagnostic X-rays, nuclear medicine, and radiation therapy are the most significant source of man-made radiation exposure to the general population. The growing use of imaging procedures has raised concerns about exposure to low-dose ionising radiation (LDIR).

Ionising radiation can induce various forms of DNA damage, including the possibility of increasing the incidence of chromosomal aberrations. While the disastrous effects of high dose ionising radiation (HDIR) is well known and accepted, the detrimental effects of low dose radiation is not well understood and has been a topic of much debate. With the advancement of high-throughput genomic technologies, global gene (mRNA) expression profiling is one of the latest approaches used to obtain information about the global cellular response to radiation.

Since skin is the first organ exposed to radiation, it is important to elucidate the cellular and molecular responses of skin cells to LDIR. However, due to ethical complications in using human skin cells for experimentation with radiation, alternate reliable biological models are in demand. Therefore, in this study we use EpiDermFT, which is a three-dimensional full thickness skin model that is composed of normal human epidermal keratinocytes (NHEK) and normal human dermal fibroblasts (NHDF). It is a reliable model since in recent years other fields of investigation such as carcinogenesis [1], [2] and wound healing [3] have been using this model in their studies. This reconstructed skin tissue is becoming an attractive in vitro model for studying the effect of radiation [4-6].

**Figure 1:**
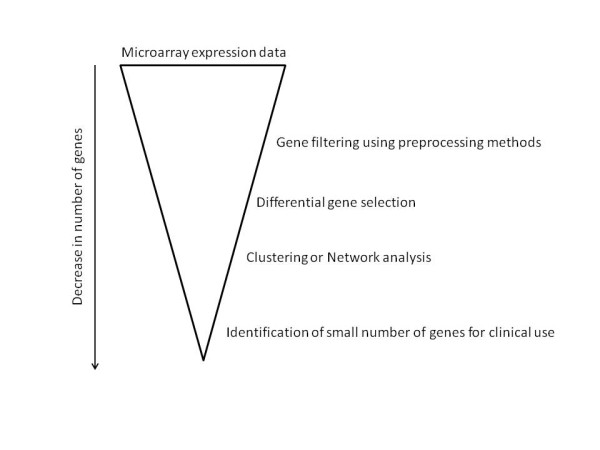
** Upside down pyramid approach of identifying key genes in a condition.** As microarray data have lots of false positives (noisy data), it is crucial that layers of gene filtering approaches be applied in order to obtain a set of few genes that may be contributing to the condition or genes that may be signatures of the condition (for clinical diagnostic purposes).

Since microarray expression data include lots of false positives, methods that allow for further gene extraction from the set of differentially expressed genes are required. As these methods are applied in a step-wise fashion the criteria also get more stringent with each step resulting in a fewer number of false positives (see Figure 1). To this end we apply the network topological analysis. Furthermore, it also aids in identifying the few genes that may be the key players in the condition.

In this study we characterised the global genomic response following 10 cGy (low dose radiation) and 100 cGy (high dose radiation) doses of X-ray ionising radiation in a tissue model by analysing the topology of gene co-expression networks. Due to massive amounts of data that get churned out via high throughput technologies such as microarrays, methods to sieve through such large amounts of data in order to identify groups of genes of interest are essential. This is what we term an üpside-down pyramid” kind of analysis. Since it is well known that not a single gene, but rather groups of genes with small changes in their expression result in a concerted global response, a systems biology approach will provide a global view of the underlying response to a certain condition or treatment. In this study, we use two graph theory methods - differential neighbourhood analysis, specifically topological/neighbourhood overlap and the difference in connectivity of genes in the coexpression networks of 10 cGy and 100 cGy - to identify interesting genes and the enriched biological processes in irradiated skin cells. The topological overlap method has been previously used to investigate the severity of Alzheimer’s disease in multiple brain regions [7,8].

If the neighbourhood of a gene in coexpression networks is representative of the ‘biological activity’ of the gene under a certain condition, then genes with high topological overlap (high overlap of their neighbours) between two dose-specific networks, may not differ greatly in their response to the two irradiation doses. However, genes with low topological overlap probably have roles/activities that are dose-specific. Many studies investigate only the genes that are unique to a condition, not common between conditions, in order to analyse how different the conditions are. However, we hypothesise that even the genes that are common between conditions (physiological, treatment or time) can contribute to the differences between conditions either by invoking different biological pathways or by invoking the same biological pathways but to varying degrees. In this study, we aimed to identify the differences between low dose and high dose ionising radiation contributed to by the genes common to both conditions by studying the gene coexpression network topology.

Studies have used the topological overlap measure for identifying metabolites that are in the same functional class [9], or for module detection, i.e. clustering of genes [10]. Other network measures have been used for other objectives as in [11-14]. While topological measures have been developed for the purposes of examining relationships among genes within the same network, our topological measure was developed to compare two different networks and use it to study the behaviour of a particular gene in separate gene coexpression networks, which represent different conditions, (i.e. different ionising radiation doses). In this manner, the measure was used as a means of computing gene coexpression network differences and then associating these differences with dose-related responses.

## Methods

### Tissue irradiation and processing

Microarray expression data was obtained from a global gene expression study aimed at assessing the genomic response to radiation conducted by Yunis et al. [15]. The details of the experimental protocol are presented there, but briefly, the effect of low versus high dose X-ray radiation on the gene expression was evaluated in a three-dimensional skin tissue model (EpiDermFT-400; MatTek Corporation, Ashland, MA). The model is reconstructed skin tissue, which is composed of keratinocytes that make up the epidermal layer, and fibroblasts that make up the dermal layer of the skin. Tissues were irradiated with 0, 10, and 100 cGy X-ray (i.e. 0, 0.1 and 1 Gy, respectively) radiation at a dose rate of 50 cGy/min using a clinical irradiator at the department of Radiation Oncology of the UC Davis Medical Center. Skin plugs were harvested at 0, 3, 8, and 24 h post irradiation. The 0 time point is about 5 min after exposure. There were 2 replicates at each time point (4 time points) and dose (3 doses) resulting in a total of 24 samples in the dataset. The integrity of the extracted RNA was verified using the 2100 Bioanalyzer (Agilent, Santa Clara, CA), and 300 ng total RNA was reverse transcribed, amplified and labeled. The resulting cRNA was hybridised to Illumina HumanRef-8 Expression Beadchips [HumanRef-8_V3_0_R2_11282963_A] and interrogated at the UC Davis Expression Analysis Core. The HumanRef-8 Expression Beadchip contains approximately 24 K well-annotated probes. All the data has been deposited in PubMed (GEO accession record GSE29344).

### Microarray data processing

Data preprocessing included the following five steps: generation of probe intensities data, data quality control, data transformation and normalisation, gene filtering and statistical analysis. The raw data, containing approximate 24 K probes and associated gene information, was processed by BeadStudio 3.4.0 along with annotation file HumanRef-8-V3-0-R2. Background subtraction of the intensities was performed in order to remove signals resulting from non-specific hybridisation. The assessment of data quality plays a crucial role in data analysis as it basically affects downstream analysis. For quality control, we used the Bioconductor packages lumi 1.2.0 [16] and arrayQualityMetrics 2.2.3 [17] to identify potential outlier samples. In our data, there were no outlier samples.

Application of standard statistical analysis techniques assumes that the data is normally distributed and has equal variance. When data violate these assumptions, appropriate data transformation is required. To satisfy the constant variance assumption, we transformed the probe intensities data (*y*) with the transeS function from Bioconductor package LMGene. This package was developed from our lab and is designed for analysis of gene expression microarray data (see LMGene package in R). It applies g-log transform (generalised log transformation) for data transformation to stabilise variance [18,19].

The g-log transformation of *y* is defined as

(1) $$g(y)=ln(y+\sqrt{y^{2}+\lambda })$$

transeS is a function of subtracting a parameter *α* prior to the g-log transformation

(2) $$g(y)=ln((y-\alpha )+\sqrt{{\left(y-\alpha \right)}^{2}+\lambda })$$

Two parameters λ and *α* can be estimated using tranest function from LMGene package. tranest is a function that uses maximum likelihood method to estimate the two parameters for the g-log transformation. LMGene was developed by our group and is similar to Limma. Limma estimates the mean square error (MSE) for each gene using a hierarchical empirical Bayes model for the collection of MSEs. LMGene provides the user with the choice of using the gene-specific MSE or the posterior MSE. The latter can be more powerful when the degrees of freedom (df) in the denominator of the F test is small, but it does entail an additional collection of assumptions. Since in this data set we have 12 df for the MSE in each gene, we chose to use the gene-specific MSE.

**Figure 2:**
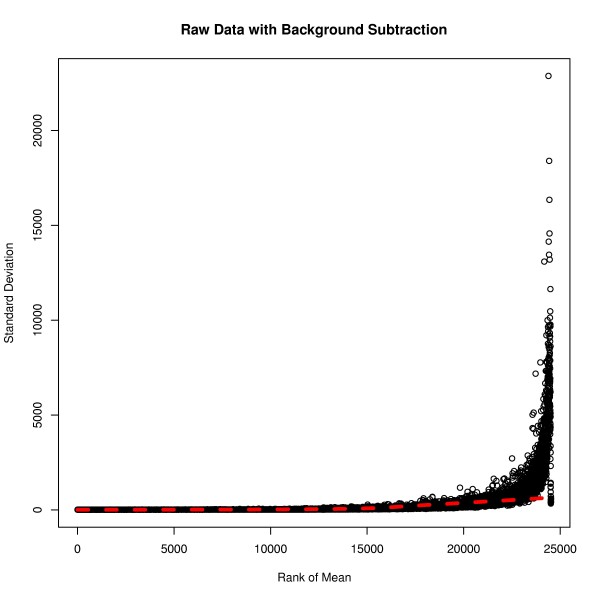
** Data preprocessing - no normalisation.** The plot of ranked mean of probe intensities versus their standard deviation for the raw data with background subtraction. The standard deviation shows a huge variation when compared to the mean intensities. The solid red line is a lowess smooth line of the probe intensities.

**Figure 3:**
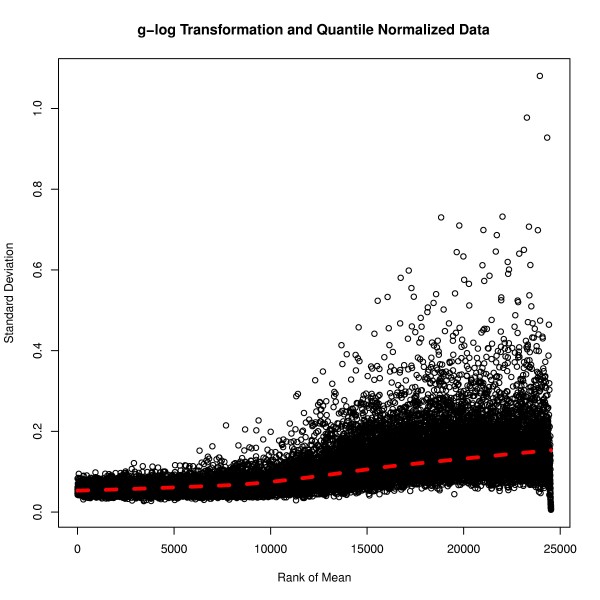
** Data preprocessing - with normalisation.** The plot of ranked mean of probe intensities versus their standard deviation for the g-log transformed and quantile normalised data. The standard deviation shows relatively constant variation when compared to the mean intensities. The solid red line is a lowess smooth line of the probe intensities.

The plot of rank mean versus standard deviation for the raw data with background subtraction shows a massive range of the standard deviation from 5.307 to 22880. As seen in Figure 2, there was enormous variation of standard deviation. After g-log transformation and quantile normalisation, the standard deviation of the transformed and normalised data has become more stable and the range significantly reduced (Figure 3). The standard deviation varied from 0.00559 to 1.081, which was a dramatic decrease compared to that of the raw data with background subtraction. Such preprocessing of the data resulted in a remarkable improvement in stabilising variance.

Noisy gene filtering prior to statistical analysis is performed to improve the power of detecting differentially expressed genes [20,21]. We checked the detection *p*-values of probes in the probe-based profile generated by BeadStudio. If a probe had a detection *p*-value > 0.05 across all that samples, then the intensities of the probe were considered unreliable and such probes were excluded from further analysis. After filtering out all probes with unreliable intensities, we were left with approximately 16 K probes that had a detection *p*-value < 0.05 in at least 1 sample. Then analysis of variance (ANOVA) model was used to detect statistically significant differentially expressed (DE) genes. Benjamini and Hochberg method was used to calculate false discovery rate (FDR) of the DE genes. For the identification of DE genes in this study, we compared gene expression profiles between 0 cGy (control) and 10 cGy (low dose radiation), and between 0 cGy and 100 cGy (high dose radiation) at each time point (0 h (control), 3 h, 8 h and 24 h).

### Coexpression networks creation and Neighbourhood analysis

For the sake of brevity, the details of the neighbourhood analysis method are only briefly described here. Please refer to [7] for a more detailed explanation. Gene coexpression networks were built by connecting genes whose pairwise expression similarity using the Pearson correlation coefficient (PCC) was at or above a threshold *t*. For two genes to be considered as coexpressed, their expression profiles need to satisfy at least one of the following conditions: (1) their correlation coefficient is higher than 0.3, and one gene is ranked as the top 3 most correlated gene of the other; (2) the correlation coefficient between them is higher than a user defined Pearson correlation coefficient threshold *t* (*t* = 0.9 in all the networks constructed here) and one gene is within the top 50 most correlated gene of the other [22]. This network method was invented by Ruan and Zhang and is described in detail in [22]. The main reason for choosing a small PCC cut-off of 0.3 is that as the PCC increases the number of nodes with no links also increases (See Figure 1 in [22]). We want a fully connected network with no isolated nodes. Also, we chose to connect the nodes with PCC of 0.3 only to 3 nodes since the median number of links is very high for nodes with a low PCC and that would result in too many unnecessary links with low PCC (See Figure 1 in [22]). According to the first condition, if 3 genes are correlated to say, gene A with correlation coefficients equal to 0.3, 0.32, and 0.4 then they get linked to gene A. On the other hand, if the 3 genes are correlated to gene A with correlation coefficients equal to 0.3, 0.28 and 0.29, then only 1 gene would get linked to gene A. However this scenario rarely, if at all, occurs in gene expression data with a correlation coefficient threshold of 0.3, hence the minimum connectivity in the networks in this paper is 3. We chose a high PCC of 0.9 in the 2nd criterion in order to have genes connected to others resulting in links between genes that have a higher possibility that they are coexpressed.

This approach resulted in a sparse, fully connected and undirected coexpression network. Minimally complex, sparse gene networks have been shown to be more robust to perturbations and may be a constraint in shaping the evolution of gene network complexity in organisms [23]. This network method has had other successful applications [7,8,24,25].

**Figure 4:**
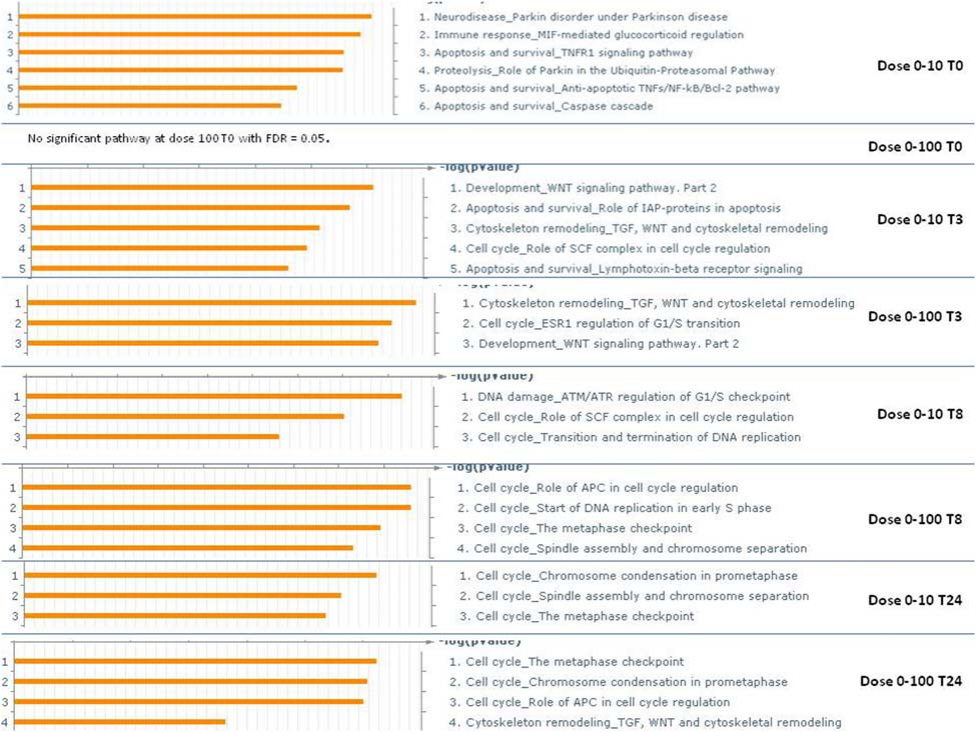
** Gene co-expression network construction.** Gene co-expression networks built from sets of differentially expressed genes. For each time point, 4 co-expression networks were built using the differentially expressed (DE) genes and the samples from that time and dose comparison. For instance, at time 0 h, the co-expression networkA was built from the expression of the 810 DE genes in the samples exposed to 10 cGy and non-exposed samples (Dose 0 cGy). Similarly, the co-expression networkB was constructed using the 525 DE genes from samples exposed to 100 cGy and non-exposed samples. Co-expression networks netAB1 and netAB2 were built using the set of DE genes common to both dose0-10 and dose0-100 comparisons but with different samples.

A coexpression network (CoExp) was constructed for an irradiation dose at a specific time, using the differentially expressed (DE) genes that were common to both doses, i.e. the intersection set. Additionally, gene coexpression networks were also created using the all the DE genes obtained by comparing irradiated and non-radiated controls in order to compare the connectivity of the common DE genes in the dose-specific networks. For a clear understanding of the networks construction refer to Table 1 and Figure 4. The coexpression network obtained from CoExp is a binary adjacency matrix with 0 referring to no link between two genes and 1 corresponding to a link between the genes. Neighbourhood analysis was performed via computing the overlap of the neighbourhoods of genes in two coexpression networks.

**Table 1:** Gene co-expression networks nomenclature and number of differentially expressed (DE) genes (nodes) used to construct them

***Networks built with DE genes from dose comparisons with control at time***		
**Dose Comparison at Time**	**No.DE genes**	**Network name (samples used to build net)**
Dose 0–10 at T0	810	networkA (samples with dose 0 and 10 cGy at T0)
Dose 0–100 at T0	525	networkB (samples with dose 0 and 100 cGy at T0)
Dose 0–10 at T3	1008	networkC (samples with dose 0 and 10 cGy at T3)
Dose 0–100 at T3	1000	networkD (samples with dose 0 and 100 cGy at T3)
Dose 0–10 at T8	203	networkE (samples with dose 0 and 10 cGy at T8)
Dose 0–100 at T8	972	networkF (samples with dose 0 and 100 cGy at T8)
Dose 0–10 at T24	702	networkG (samples with dose 0 and 10 cGy at T24)
Dose 0–100 at T24	953	networkH (samples with dose 0 and 100 cGy at T24)
***Networks built with common DE genes from two dose comparisons with control at time***		
**Dose Comparison at Time**	**No.common DE genes**	**Network name (samples used to build net)**
Dose 0–10 at T0	199	netAB1 (samples with dose 0 and 10 cGy at T0)
Dose 0–100 at T0		netAB2 (samples with dose 0 and 100 cGy at T0)
Dose 0–10 at T3	503	netCD1 (samples with dose 0 and 10 cGy at T3)
Dose 0–100 at T3		netCD2 (samples with dose 0 and 100 cGy at T3)
Dose 0–10 at T8	86	netEF1 (samples with dose 0 and 10 cGy at T8)
Dose 0–100 at T8		netEF2 (samples with dose 0 and 100 cGy at T8)
Dose 0–10 at T24	482	netGH1 (samples with dose 0 and 10 cGy at T24)
Dose 0–100 at T24		netGH2 (samples with dose 0 and 100 cGy at T24)

In networkA (number of nodes = 810) inter-gene correlation was computed for samples exposed to dose 0 and 10 cGy at time 0. In networkB (number of nodes = 525) inter-gene correlation was computed for samples exposed to dose 0 and 100 cGy at time 0. In networkC inter-gene correlation was computed for samples exposed to dose 0 and 10 cGy at time 3. In networkD inter-gene correlation was computed for samples exposed to dose 0 and 100 cGy at time 3. Same protocol for networks networkE,networkF, networkG and networkH. In netAB1 (number of nodes = 199) inter-gene correlation was computed for samples exposed to dose 0 and 10 cGy at time 0. Same protocol for networks netAB2, netCD1, netCD2, netEF1, netEF2, netGH1 and netGH2. The main difference between networks in the top half and the bottom half of the table is the nodes/DE genes used to construct them.

Let two coexpression networks built from the intersection of DE genes be referred to as *network1* and *network2* corresponding to dose 1 and dose 2 at time T1, respectively. Since *network1* and *network2* were built using the intersection genes between doses 1 and 2, the nodes in both the networks are the same, although the connections among them are different. Let each node/gene in the network be denoted as *gene*_*i*_ where *i* = 1,2,…,*m* and *m* is the total number of nodes in the network. Topological overlap between *network1* and *network2* refers to the overlap of the genes connected to *gene*_*i*_ in *network1* and *network2*, i.e. overlap of the neighbourhoods of *gene*_*i*_. Let the genes connected to a *gene*_*i*_ in *network1* be referred to as *X* and those in *network2* be referred to as *Y*. The connectivity or degree of *gene*_*i*_ in *network1* is *d*1_*i*_ and that in *network2* is *d*1_*i*_. The topological overlap (TO) for *gene*_*i*_ between *network1* and *network2* is given by

(3) $$TO_{gene_{i}}=\frac{\left|X\cap Y\right|}{max(d1_{i},d2_{i})}$$

The value of TO of any gene ranges from 0 to 1, with 1 indicating maximum overlap of its neighbourhoods in both networks. In gene coexpression networks, the maximum value of TO will, more often than not, be less than 1. Further details of this method are presented in [7].

In this analysis, genes with a topological overlap of ≤ 0.1 between two networks were selected for further analyses since this resulted in less than 10% of the genes in the network being selected under this criteria. Genes with other values of topological overlap, if properly justified, can also be considered. Comparisons against 1000 random networks (random additions or deletion of links to the original network while keeping the degree of the genes equal to the original network) using *t*-statistics were made to assess the significance of the genes with TO = 0.1.

Biological analysis and interpretation of genes of interest was performed by identifying significant biological processes or pathways. Statistically significant biological pathways were identified using the well annotated GeneGo MetaCore^*TM*^ database [26], which is a commercial tool. MetaCore^*TM*^ is based on a proprietary manually curated database of human protein-protein, protein-DNA and protein compound interactions, metabolic and signalling pathways and the effects of bioactive molecules in gene expression [26]. We upload a set of genes into GeneGo MetaCore module and GeneGo checks to find which pathways are significant with a FDR =0.05 (default setting). It compares the user’s uploaded set of genes with the set of genes/proteins stored in their Pathways Database. Significance (p-values) in GeneGo of a biological pathway is evaluated based on the size of the intersection between user’s dataset and set of genes/proteins corresponding to a network module/pathway in question. Thorough details of the significance calculation is provided in [27,28].

## Results and discussion

### Global gene expression profiles

Table 2 shows the number of differentially expressed (DE) genes for each dose comparison with control dose 0 cGy, at a specific time point as well as the common DE genes between dose comparisons (DE genes provided in Additional file 1).

**Table 2:** Number of dose-dependent differentially expressed (DE) genes

**Dose Comparison**	**Time (hr)**	**#DE genes** (FDR = 10%)	**#common DE genes**	**% common genes**
Dose 0–10	T0	810	199	17.5
Dose 0–100		525		
Dose 0–10	T3	1008	503	33.4
Dose 0–100		1000		
Dose 0–10	T8	203	86	7.9
Dose 0–100		972		
Dose 0–10	T24	702	482	41.1
Dose 0–100		953		

Percentage of common DE genes is given by $\frac{numberofcommonDEgenes}{\left(Dose1comparisonDEgenes+Dose2comparisonDEgenes-numberofcommonDEgenes\right)}\times 100$

**Figure 5:**
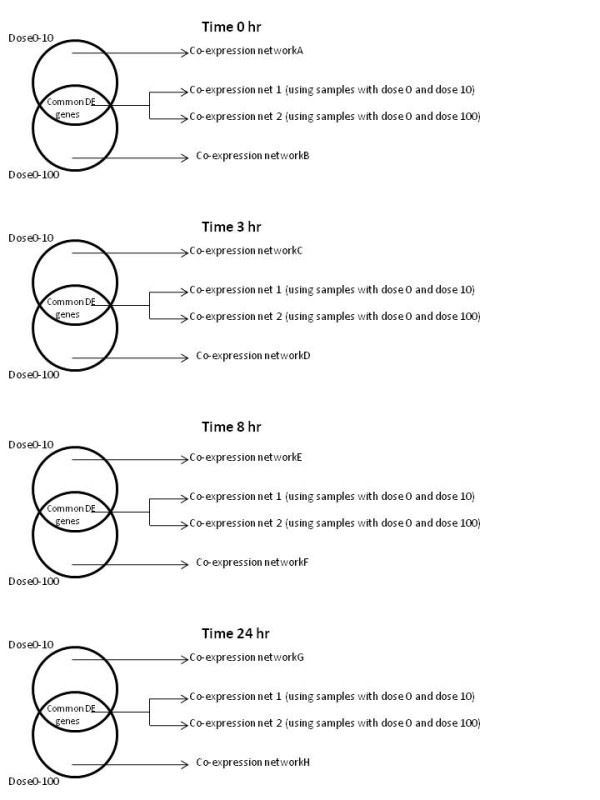
** Top few statistically significant (FDR = 0.05) GO processes of the DE genes of each dose comparison at each time point.** Left side of the figure shows the significant processes and the right side shows the comparison performed. No statistical significant processes was identified for the dose0-100 time 0 comparison.

As can be seen, the number of DE genes change over time. We observed that while dose 100 cGy perturbed fewer genes than dose 10 cGy initially, over time the number of perturbed genes increased and remained almost the same. Meanwhile, the response to dose 10 cGy in terms of number of perturbed genes greatly varied with time. Furthermore, the difference in the number of perturbed genes due to the two doses was the largest at time 0 and 8 h. Top GO processes of the DE genes at each dose and time comparison is shown in Figure 5.

An interesting observation that can be noted from Table 2 is that the percentage of common DE genes in each of the 4 scenarios were quite large. Furthermore, the percentage of common DE genes within each set of DE genes is also quite large. For instance, the set of 482 common genes at T24 is 68.7% of the set of 702 DE genes obtained from comparing dose 0 and dose 10 cGy at T24. This indicates that there are many similar responses to the low dose and high dose radiation. When analysis of the biological processes of the unique genes (all DE genes minus the common DE genes for each comparison) was carried out for each dose comparison at each time point, a wide range of cellular processes were present (data not shown). It was not possible to highlight the differences in response between the high and low dose based on the unique genes alone.

Since microarray expression data include lots of false positives, methods that allow for further gene extraction from the set of differentially expressed genes are required. As these methods are applied in a step-wise fashion the criteria also get more stringent with each step resulting in a fewer number of false positives. To this end we apply the network topological analysis.

Figure 4 illustrates the way in which different dose specific coexpression networks were built at each time point and the genes/nodes used in their creation. Table 1 shows the nomenclature of the gene coexpression networks and the number of DE genes (nodes) used to construct them.

When we analysed the common DE genes between the dose 0–10 cGy and dose 0–100 cGy comparisons at time 0 h (5 min post radiation), we found a wide range of biological processes perturbed, but with no specific processes dominating. We attributed this phenomenon to the fact that the system may have been going through initial shock rather than responding to the specific irradiation doses. Furthermore, if it was going through shock *and* responding to radiation dose, it is difficult to differentiate the two effects, i.e. data has low power to distinguish these effects. Therefore, we carried out detailed analysis on time points 3, 8 and 24 h when the expression patterns of the genes were more likely responding to the radiation dose. However, it is interesting to note that dose 10 cGy perturbed a greater number of genes compared to dose 100 cGy at time 0 h, an observation that needs further investigation. Similar effect was also observed in other LDIR studies [29,30]. One possible explanation for this could be that cells are quicker to respond to the low dose radiation as they are not as ßtunned” as the cells exposed to the high dose. Once recovered from the exposure, the high dose irradiated cells have high numbers of perturbed genes.

### Genes with low topological overlap between irradiation dose networks

**Figure 6:**
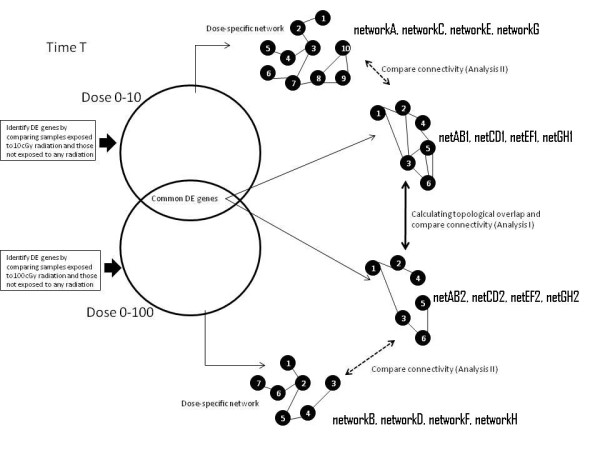
** Analysis strategy.** Analysis strategy for comparing dose-specific gene co-expression networks for topological overlap and connectivity differences. Each network refers to a particular dose-specific network at a particular time point. Analysis I refers to comparing topological overlap and connectivity differences between networks built with common DE genes. In Analysis I, netAB1 is compared with netAB2, netCD1 with net CD2, netEF1 with netEF2 and netGH1 with netGH2. Analysis II refers to comparing connectivity differences only. In this step, networkA is compared to networkB (In order to keep the picture clear, we did not put an analysis arrow between networkA and networkB across the picture) to identify genes with high connectivity difference and the selected genes are also investigated in the comparison between netAB1 and netAB2. This kind of analysis is carried out for all the networks in a similar fashion.

We computed the topological overlap (TO) (see Methods) of each gene between two dose-specific coexpression networks built with the set of common DE genes - for instance compared netCD1 with netCD2; netEF1 with netEF2; and netGH1 with netGH2 (see Figure 6 - Analysis I).

The motivation behind the low topological overlap idea is that genes with low topological overlap (low TO genes) may be performing different activities/roles or are a part of different biological processes in the two networks. Therefore, we investigated the neighbours of the low TO genes. ‘Neighbours’ of the low TO genes refer to the genes directly connected to the gene that has a low topological overlap between two networks. We identified the enriched biological processes of the neighbours of some of these low TO genes to understand what processes were changing across doses.

**Figure 7:**
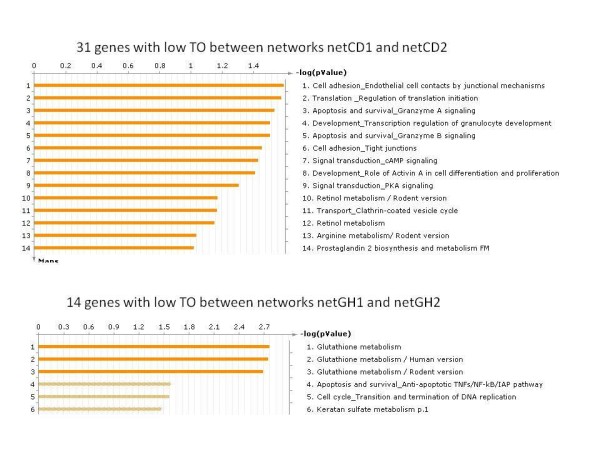
** Low topological overlap genes.** The GO processes of the 31 genes with low topological overlap (TO ≤ 0.1) between networks netCD1 and netCD2 and 14 genes between networks netGH1 and netGH2.

We investigated genes with a TO value ≤ 0.1. These genes were considered to have ‘low TO’ between two networks. There were 31 genes (6.2%) with a TO ≤ 0.1 between nets netCD1 and netCD2 and 14 (2.3%) genes with a TO ≤ 0.1 between nets netGH1 and netGH2 (Figure 7). As can be seen, choosing a threshold of 0.1 resulted in less than 10% of the genes in the network being selected using this criteria. Since the conditions are similar (both are exposure to radiation; 50% of the samples (dose 0) are the same across two networks (see Table 1); and high overlap of the common DE genes - Table 2, 4th column) the two networks cannot be very different. However, there would be a small number of genes contributing to the difference between the networks since the conditions are not identical. Since the system used in this study is a skin model made of keratinocytes and fibroblasts and, although it is a good model to study human skin, it is not the complete skin model (with nerves, blood vessels, glands, etc.) that exists in real human skin. Therefore, there is bound to be more similarities between the different networks than differences. The statistical significance (*p* value) of these genes with TO = 0.1 was *p* < 0.05. There were no genes with TO ≤ 0.1 with a statistical significance of *p* < 0.05 between networks netEF1 and netEF2. This was due to the small size of the network.

The biological processes of the 31 genes which had a low TO between netCD1 and netCD2 were analysed (see Additional file 2). Endothelial cell contacts by junctional mechanisms (*p* =2.5e-2), regulation of translation initiation (*p* =2.6e-2), granzyme A signalling (*p* =2.9e-2), granzyme B signalling (*p* =3.1e-2), etc. emerged as significant processes. Granzymes are serine proteases that are released by cytoplasmic granules within cytotoxic T cells and natural killer cells and induce apoptosis [31]. Granzyme B is responsible for the rapid induction of caspase-dependent apoptosis [31]. Granzyme-A-induced cell death is mainly characterised by the generation of single-stranded DNA nicks [31]. The significant biological processes of the 14 genes, which had low TO between netGH1 and netGH2, included glutathione metabolism (*p* =1.7e-3), anti-apoptotic TNFs/NF-KB/IAP pathway (*p* =2.6e-2), cell cycle - transition and termination of DNA replication (*p* =2.7e-2), keratan sulphate metabolism, (*p* =3.4e-2). Studies have shown altered glutathione levels in cells exposed to ionising radiation [32,33]. Therefore, finding glutathione metabolism as one of the highly affected processes at 24 h across the two doses is interesting and should be further investigated.

We also checked to see which biological pathways were differentially affected between the set of 31 genes and 14 genes, i.e. biological pathways that were differentially affected at time 3 h and 24 h. Glutathione metabolism emerged as the significant pathway (FDR = 0.05). Glutathione has been known to play an important role in antioxidant defense, nutrient metabolism, and regulation of cellular events [34]. It’s metabolism has been associated with both protective and pathogenic roles in cancer [35].

Of the 31 low TO genes between netCD1 and netCD2, 13 genes were downregulated at both doses and 17 genes were upregulated at both doses. However, 1 gene - Sorbin and SH3 domain-containing protein 1 (SORBS1) was downregulated at dose 10 cGy and upregulated at dose 100 cGy. Of the 14 low TO genes between netGH1 and netGH2, 9 were downregulated at both doses and 5 were upregulated at both doses. Of interest was tumor necrosis factor receptor superfamily, member 1B (TNFRSF1B) which was upregulated. Studies have suggested a role of TNFRSF1B in protecting neurons from apoptosis by stimulating antioxidative pathways [36]. The TNF superfamily has been a rich source for drug targets in inflammatory and autoimmune diseases as well as cancer [36].

We imported the list of 31 and 14 genes into DAVID (http://david.abcc.ncifcrf.gov) to identify the association of these genes with any known diseases/conditions. It was interesting to note that the list of 31 genes is linked to immune, cardiovascular, developmental, cancer and metabolic conditions (see Additional file 3). The set of 14 genes is linked to cancer, ageing, cardiovascular, immune, and metabolic among other conditions (see Additional file 4). This shows that these genes are important and play a role in radiation response.

### Connectivity difference between irradiation dose networks built with common DE genes

We also investigated the difference in the number of network connections (edges or links in the co-expression network) of the common DE genes between the two networks built for each dose comparison at a specific time (see Figure 6 - Analysis I). Our hypothesis is that genes with a high connectivity difference are ‘more active’ or ‘over expressed’ at a specific dose and time, thereby activating certain biological processes. We defined a ‘high’ difference in connectivity if a gene had a difference of ≥ 10 connections between dose 0–10 cGy and dose 0–100 cGy networks at a specific time point.

**Figure 8:**
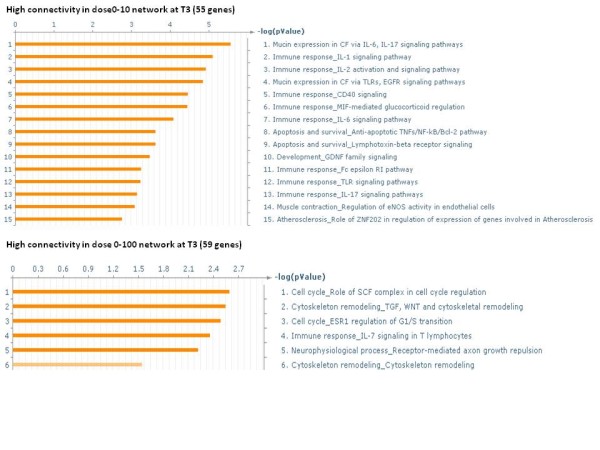
** Significant biological processes of genes with large connectivity differences between netCD1 and netCD2 at 3 h.** Significant processes from GeneGo of the genes with high difference (≥ 10 connections) in connectivity between networks netCD1 and netCD2. The number in parentheses is the number of genes having a higher connectivity in that network. The FDR of the biological processes of the 55 genes having a greater number of connections in dose0-10 cGy network is 0.05 and that of the 59 genes with greater connections in dose0-100 cGy network is 0.1.

At 3 h there were 114 genes that had a high difference in connectivity between dose 0–10 cGy and dose 0–100 cGy networks. There were 55 genes with a higher connectivity in dose 0-10 cGy network and 59 genes with a higher connectivity in dose 0–100 cGy network (See Additional file 5). The significant biological processes of the 55 and 59 genes are shown in Figure 8. It can be seen that while immune response was the major response in the cells exposed to dose 10 cGy, cell cycle processes were dominating at dose 100 cGy.

**Figure 9:**
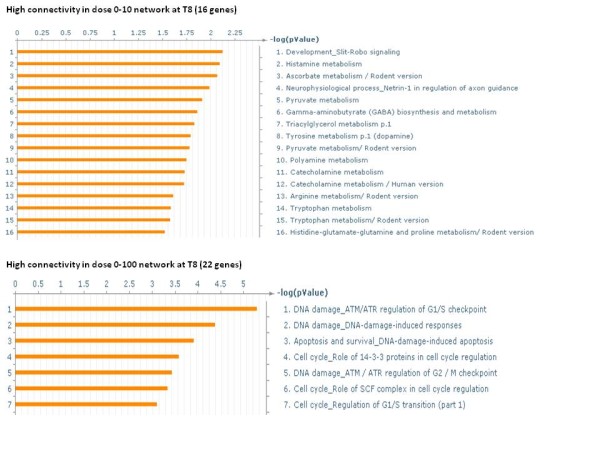
** Significant biological processes of genes with large connectivity differences between netEF1 and netEF2 at 8 h.** Significant processes from GeneGo of the genes with high difference (≥ 10 connections) in connectivity between networks netEF1 and netEF2. The number in parentheses is the number of genes having a higher connectivity in that network. The FDR of the biological processes of the 16 genes having a greater number of connections in dose0-10 cGy network is 0.05 and that of the 22 genes with greater connections in dose0-100 cGy network is 0.05.

At 8 h there were 38 genes with a high connectivity difference between dose 0–10 cGy and dose 0–100 cGy networks (See Additional file 6). The significant biological processes of the genes with higher connectivity in the individual networks is shown in Figure 9. While metabolism related processes were prominent in cells exposed to dose 10 cGy and DNA damage induced processes were active in cells exposed to 100 cGy.

**Figure 10:**
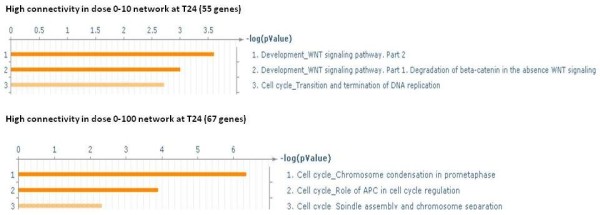
** Significant biological processes of genes with large connectivity differences between netGH1 and netGH2 at 24 h.** Significant processes from GeneGo of the genes with high difference (≥ 10 connections) in connectivity between networks netGH1 and netGH2. The number in parentheses is the number of genes having a higher connectivity in that network. The FDR of the biological processes of the 55 genes having a greater number of connections in dose0-10 cGy network is 0.05 and that of the 67 genes with greater connections in dose0-100 cGy network is 0.05.

At 24 h there were 122 genes with a high connectivity difference between dose 0–10 cGy and dose 0–100 cGy networks and their biological processes are shown in Figure 10 (See Additional file 7). It was interesting to note that at 24 h, processes involved with Wnt signalling was beginning to overshadow other processes in cells exposed to 10 cGy radiation while cell cycle processes prevailed in cells exposed to 100 cGy radiation. The Wnt signalling pathway has been associated with carcinogenesis and development [37-39].

**Figure 11:**
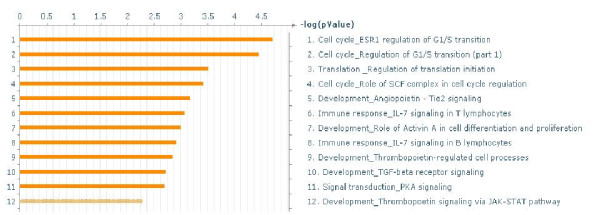
** Statistically significant biological processes across time and dose.** Topological analysis showed what processes were more dominant at each time and dose.

Figure 11 shows the change over time and dose in terms of different biological processes being dominant. This clear highlighting of the different sets of processes active at the different doses is due to the application of topological methods (see Figures 5, 8, 9, 10). Our results indicate that at low dose the cells are involved in adaptive responses while at the high dose, they are mostly involved in cell cycle processes.

### Comparison with Voy et al. analysis

A report by Voy et. al has used a systems biology approach for the analysis of the effects of low dose ionising (10 cGy) radiation in mice spleen [40]. Voy et. al identify sets of co-expressed genes by identifying ‘cliques’ in co-expression networks.

\Voy and colleagues harvested their tissue at 3.5 h and exposed the tissue to 10 cGy ionising radiation. We compared our microarray results obtained at 3 h to theirs. Despite the difference in the kind of tissue and model between the two studies, we found many similarities in the biological processes. In our study we noted that immune response was the predominant response at dose 10 cGy at 3 h. Voy et. al also concluded that immune response pathways were highly activated at dose 10 cGy at 3.5 h. Furthermore, some genes like Notch, IL, Cyp, Stat, Tmem, etc. were found in both studies. We also found several transmembrane (Tmem-) genes which were also identified in their study and hypothesised to be involved in ionising radiation response. Studies like ours and that of Voy et. al are complimentary, as well as help in providing support for the results obtained using sophisticated computational methods such as ours in studies relating to LDIR.

### Connectivity difference of genes between the common DE genes network and all DE genes network

Networks networkA, networkB, networkC, networkD, networkE, networkF, networkG and networkH were built from all the DE genes obtained by comparing the gene expression between either dose 10 cGy or 100 cGy with control dose 0 cGy at different time points (see Table 1). In this section, we analyse the connectivity of the common DE genes (Table 1, lower half) within the networks built with all the DE genes (Table 1, upper half). This idea is illustrated in Figure 6 - Analysis II. For example, the connectivity of 503 genes that are common to the sets of DE genes obtained by comparing dose 0 cGy with dose 10 cGy and dose 0 cGy with dose 100 cGy will be investigated in networks networkC, networkD, netCD1 and netCD2. Genes with a difference in the number of connections/links/edges ≥ *T*, where *T* is some threshold, are analysed further for biological relevance. For this study, we chose *T* = 10.

**Figure 12:**
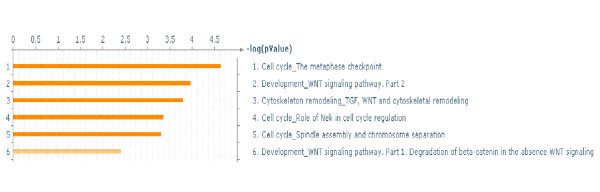
** Significant biological processes of genes with large connectivity differences between networkC and networkD at 3 h.** Significant processes (FDR = 0.05) of the 105 genes that had ≥ 10 network connections difference between networks networkC and networkD.

Among the 503 common DE genes (between netCD1 and netCD2), 105 genes had a connectivity difference ≥10 between networkC and networkD, whereas 29 genes had a connectivity difference ≥10 between networkC and networkD, *and* between netCD1 and netCD2. The set of 29 genes included genes like Cyclin G2 (CCNG2), CAMP responsive element binding protein 3-LIKE 2 (CREB3L2), eukaryotic translation initiation factor 3, subunit D (eIF3d), forkhead box O1 (FOXO1A), myeloid/lymphoid or mixed-lineage leukemia (MLLT11), protein phosphatase 2, regulatory subunit B, gamma isoform (PPP2R2C). Among the 105 genes were genes linked to breast cancer, such as retinoblastoma 1, cyclin-dependent kinase inhibitor 1a, etc. This set also included genes like cyclin-dependent kinase inhibitor 1A (CDKN1A), FYN oncogene related to SRC, FGR, YES (FYN), G protein-coupled receptor, family C, group 5, member A (GPRC5A), mastermind-like 1 (MAML1), MAX dimerisation protein 1 (MXD1), membrane metallo-endopeptidase (MME), transcription factor 20 (TCF20), tumor protein p53 inducible nuclear protein 1 (TP53INP1), etc. The significant biological processes of the 105 genes (See Additional file 8) is shown in Figure 12.

Of the 86 common DE genes (between netEF1 and EF2), 10 genes had a connectivity difference ≥10 between networkE and networkF, *and* between netEF1 and netEF2, whereas there were 28 genes with a connectivity difference ≥10 only between networkE and networkF networks (See Additional file 9). Furthermore, the breast cancer 1, early onset (BRCA1) gene was one of the 10 genes. Numerous studies have BRCA1 is crucial for maintaining genomic stability, DNA repair and is a tumour suppressor [41,42]. It had greater number of connections in the dose 0–10 cGy network (networkE). Cyclin-dependent kinase inhibitor 1A (CDKN1A), homeobox D13 (HOXD13), interferon regulatory factor 2 binding protein 2 (IRF2BP2), ribonucleotide reductase M2 (RRM2), UDP-N-acetyl-alpha-D-galactosamine polypeptide N-acetylgalactosaminyltransferase 3 (GALNT3), etc. were among the 28 genes. The set of 28 genes was too small to identify statistically significant processes, however, the genes were mainly involved in metabolism and Pol II transcription.

**Figure 13:**
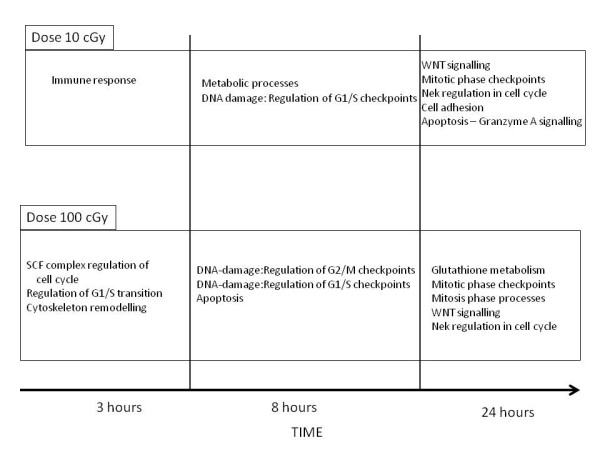
** Significant biological processes of genes with large connectivity differences between networkG and networkH at 24 h.** Significant processes (FDR = 0.05) of the 99 genes that had ≥ 10 network connections difference between networks networkG and networkH.

99 genes out of the 482 common DE genes had a connectivity difference ≥ 10 between networkG and networkH (See Additional file 8). Aurora kinase A (AURKA), centromere protein A (CENPA), CHK1 checkpoint homolog (CHEK1), chromodomain helicase DNA binding protein 1-like (CHD1L), SCL/TAL1 interrupting locus (STIL), etc. were among the genes present in the set of the 99 genes. The majority of these genes play a role at the G2/M phase of the cell cycle. The significant biological processes of the 99 genes (See Additional file 10) is shown in Figure 13.

## Conclusions

Ionising radiation is a proven human carcinogen. Most studies have analysed the effects of high dose radiation such as atomic bomb survivors in Japan, people exposed during the Chernobyl nuclear accident, patients undergoing radiation therapy, uranium miners, etc. However, it has been difficult to measure and assess the risk of cancer in people exposed to lower doses of ionising radiation such as the people living at high altitudes, who are exposed to more natural background radiation from cosmic rays than people at sea level. In our study, one of the points of interest was that as time progressed and even though the biological processes were very different across doses at 3 h, the response - by the set of common DE genes- to both 10 cGy (low) and 100 cGy (high) doses of X-ray radiation was similar in terms of the biological pathways (mostly cell cycle processes) affected at 24 h (Figure 11). Most of the processes at 24 h under both doses are associated with mitosis checkpoints. We have shown that the set of common DE genes account for a large percentage of the DE genes at each comparison. Therefore we claim that even 10 cGy radiation is not ‘low’ when analysed from a biological processes viewpoint. It is possible that cells exposed to the LDIR have better survival compared to the cells exposed to the HDIR after 24 h, despite the similarity in the processes at 24 h. It would be worth investigating this idea. It must be noted that our results may be specific to the model used in our study, but comparison to other LDIR studies have shown similar results in terms of the biological processes at each time and dose [29,30,43].

In this analysis we applied systems biology network analysis methods to study the difference in the effects of two doses of radiation on skin cells at different time points. This computational method of analysis helps to identify transcriptional differences between two very similar conditions. Our method of analysis is a way of fine tuning down to the sets of genes that may be key players at a certain time and, hence, by investigating the biological pathways of those key genes, we were able to identify the dominating processes at each time point. So it is the difference between coarse tuning - using DE gene selection or clustering and then identifying biological pathways technique - and fine tuning - our method of analysis via topological analyses.

Eukaryotic cells have many biological mechanisms to identify and repair damaged DNA to preserve genomic integrity. These mechanisms include the activation checkpoints and induction of cell cycle arrest, to allow the cell time to repair the damage. Cell cycle arrest can be triggered at G1/S, intra-S and G2/M phases. We saw these processes being dominant at various dose and time points in Figure 11. Microarray expression analysis of skin cells under normal conditions would probably show similar biological processes over time as cell cycle activity in skin cells is very high. There aren’t any reports of microarray expression of normal skin cells over time, to the best of our knowledge, to verify this idea. It is due to topological analyses that we were able to extract genes which have been reported to be highly associated with cancer, such as BRCA1, ageing, immune response, etc., which would not have been present under normal conditions. Methods like ours can help in generating testable hypotheses while dealing with high throughput data.

There has been a report by Voy et. al that has used a systems biology approach for analysing the effects of low dose ionising (10 cGy) radiation [40]. Voy et. al identify sets of co-expressed genes by identifying ‘cliques’ in co-expression networks. To the best of our knowledge, Voy et. al is the only group, other than ours, that has analysed LDIR from a networks perspective. Not only is our experimental design very different but our computational analysis is also different. Our network topological analyses seek to identify the differences between two radiation doses networks, i.e. identify the differences across very similar conditions. We consider 10 cGy and 100 cGy ‘similar’ in the sense that both are ionising xradiation and the conditions are similar although the magnitudes are different. Furthermore, we use only local network properties to distinguish between the doses. The reason for this is that global network characteristics of very similar conditions (represented as networks) do not have enough power to distinguish between conditions. For example, consider two oranges - one sweet and one sour. Both look the same in terms of structure and texture, however, they differ in taste. If we built a network to represent the sweet orange and the sour orange, the global network characteristics, such as network density, mean network connectivity, etc. would be similar between the two orange networks. However, since there is a difference in taste, there will be some differences between these two networks which would be local network properties. Therefore, we do not use global properties to identify the differences between dose 10 cGy and 100 cGy in this manuscript. Other reports have also shown how global properties cannot distinguish between very similar networks [7,44].

Systems biology methods like ours are in high demand as the differences between many conditions, be they neurodegenerative diseases, brain diseases, different kinds of cancers, different degrees of disease severity, etc., are very subtle and cannot be easily highlighted using the usual off-the-shelf clustering or biological pathways identification algorithms. Comparing networks of any kind - social, biological, etc. - is a difficult and ongoing field of research. Comparisons of gene coexpression networks by identifying hub genes or by looking at the neighbourhoods of gene in networks that share few common genes and have many different genes between them (both are cases where there are very obvious differences between networks that can be detected using simple methods), has been previously reported. However, comparing different networks representing very similar conditions and trying to identify the differences between them is a difficult task. We have presented the application of a method that can aid in this task.

## Competing interests

The authors have no competing of interest.

## Authors’ contributions

RY and DMR conceived of the experimental study. RY isolated the RNA and sent it to the UC Davis Microarray core for hybridisation. Shiquan Wu and XC carried out microarray preprocessing steps such as variance stabilisation, normalisation and identification of statistically significant differentially expressed genes. MR constructed the co-expression networks, developed the topological overlap method, and performed the analyses of the networks. MR carried out the biological interpretation of results. All authors read and approved the final manuscript.

## Supplementary Material

Additional file 1 Differentially expressed genes between 0 cGy (control) and 10 cGy, and between 0 cGy (control) and 100 cGy at time 3 h, 8 h and 24 h. Click here for file

Additional file 2 31 low topological overlap genes between netCD1 and netCD2 and 14 low topological overlap genes between netGH1 and netGH2. Click here for file

Additional file 3 Association of 31 genes (low TO at 3 h) to medical conditions. Click here for file

Additional file 4 Association of 14 genes (low TO at 24 h) to medical conditions. Click here for file

Additional file 5 Genes with large connectivity differences between netCD1 and netCD2 at 3 h. Click here for file

Additional file 6 Genes with large connectivity differences between netEF1 and netEF2 at 8 h. Click here for file

Additional file 7 Genes with large connectivity differences between netGH1 and netGH2 at 24 h. Click here for file

Additional file 8 Genes with large connectivity differences between networkC and networkD at 3 h. Click here for file

Additional file 9 Genes with large connectivity differences between networkE and networkF at 8 h. Click here for file

Additional file 10 Genes with large connectivity differences between networkG and networkH at 24 h. Click here for file
