# Supplementary figures and images for: Comparison of low and high dose ionising radiation using topological analysis of gene coexpression networks
Source: BMC Genomics. 2012 May 17;13:190. doi: 10.1186/1471-2164-13-190 (PMC3443446; doi:10.1186/1471-2164-13-190)

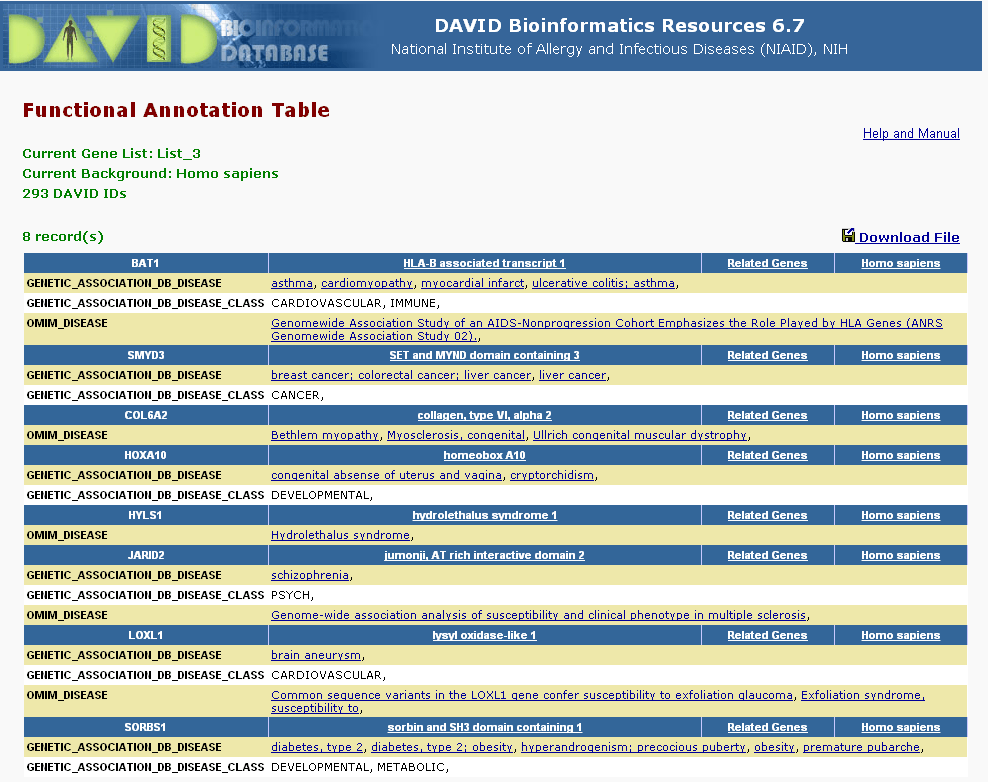

Supplement: Additional file 3 — Association of 31 genes (low TO at 3 h) to medical conditions. [file 1471-2164-13-190-S3.gif]

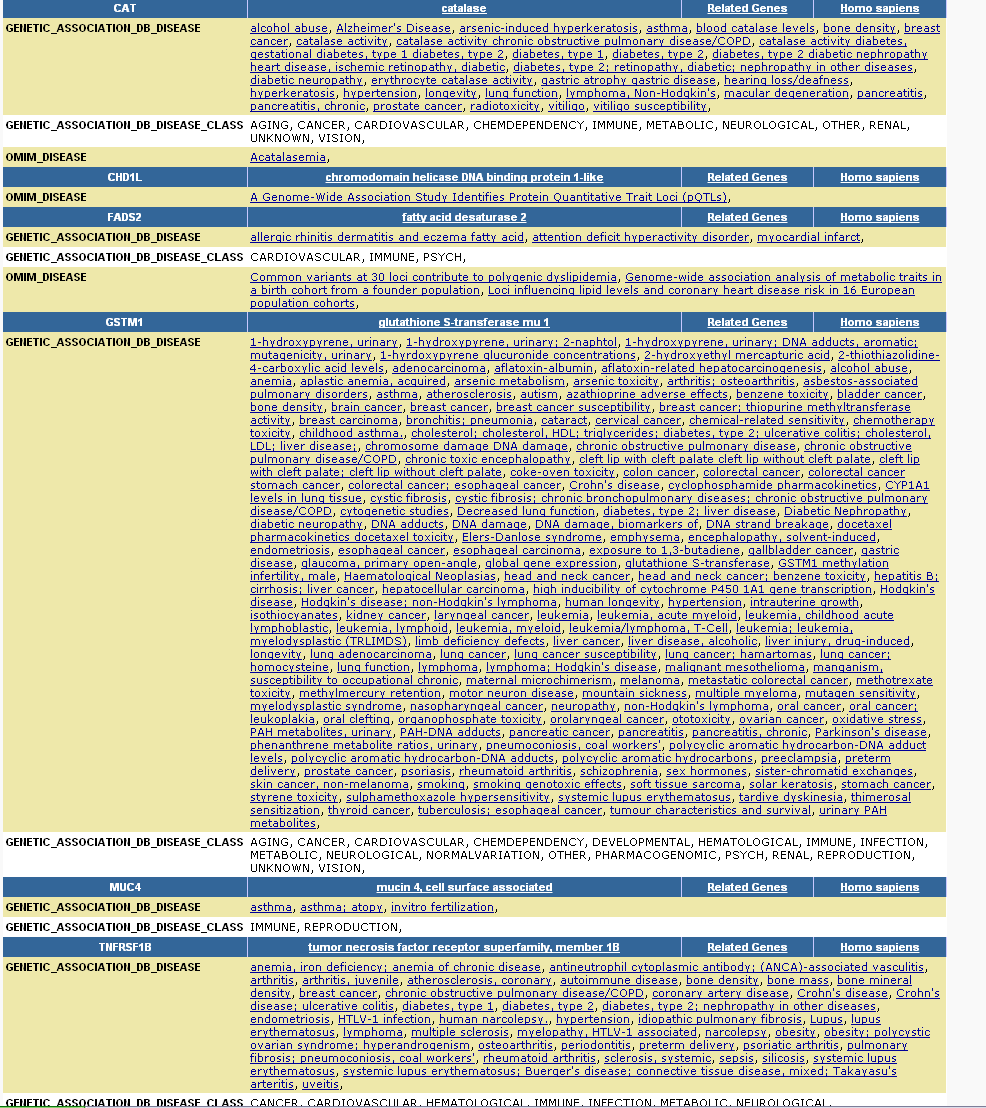

Supplement: Additional file 4 — Association of 14 genes (low TO at 24 h) to medical conditions. [file 1471-2164-13-190-S4.gif]
